# Delayed diagnosis of blunt thoracic aortic injury due to thoracic vertebral fracture: A case report and literature review

**DOI:** 10.3389/fsurg.2022.980451

**Published:** 2022-10-20

**Authors:** Xinhuan Lei, Zhenghua Hong, Weixing Pan, Jie Xiang, Hua Luo

**Affiliations:** ^1^ Department of Orthopedic, Taizhou Hospital of Zhejiang Province Affiliated to Wenzhou Medical University, Taizhou, China; ^2^ Department of Radiology, Taizhou Hospital of Zhejiang Province Affiliated to Wenzhou Medical University, Taizhou, China

**Keywords:** thoracic aortic injury, thoracic vertebral fracture, thoracic endovascular aortic repair, delayed diagnosis, case report

## Abstract

Blunt vascular injury of the aorta combined with thoracolumbar fracture is rare. Delayed diagnosis may have a catastrophic outcome. We present a case of blunt thoracic aortic injury combined with a vertebral body fracture at T10 after a fall from height in which the diagnosis was delayed. After consultation with the vascular and spinal surgeons, we performed a thoracic endovascular aortic repair. When the patient’s condition had stabilized, the fractures were reduced using posterior vertebral instrumentation. Prolonged compression of the thoracic aorta resulted in extensive necrosis of muscle tissues in the right lower leg. Fortunately, clinical and radiological examinations performed 7 months and 1 year later did not reveal any further damage. Cases of thoracic vertebral fracture with concomitant blunt thoracic aortic injury reported in the literature are reviewed. Thoracic endovascular aortic repair is a feasible, safe, and effective minimally invasive treatment for aortic injury when combined with thoracic vertebral fracture.

## Introduction

Traumatic thoracic fracture presenting with blunt vascular injury is uncommon (1). This type of injury may be asymptomatic or very serious, with a mortality rate of up to 90% in the event of full laceration of the vessel (2, 3). Delayed diagnosis and treatment may have a catastrophic outcome. Furthermore, a conventional surgical approach might be highly invasive and take too long to perform. In this report, we describe a case of delayed diagnosis of blunt thoracic aortic injury that occurred when the vessel became trapped for 2 days within a fractured thoracic vertebra and was treated successfully by thoracic endovascular aortic repair (TEVAR) and subsequent posterior transpedicle screw fixation. The EMBASE, PubMed, and Web of Science databases were searched using the terms “blunt thoracic aortic injury” and “thoracic fracture” to identify reports of blunt thoracic aortic injury as a result of thoracic fracture. The details of the reported identified (2, 4–11) are shown in Table 1.

**Table 1:** Summary of 9 cases of aortic injury combined with thoracolumbar fracture identified by a literature search with addition of our case.

Author/year	Age	Sex	Vascular lesion		Spinal fracture		Initial status	Surgical treatment		Outcome
			Type	Site	Level	Type	ASIA (level)	Vascular	Spinal	ASIA (level)
Poelaert et al. 1995	32	M	IT	Thoracic and abdominal aorta	T10	NR	E	Y	Y	E
Murakami et al. 1998	21	M	P	Thoracic and abdominal aorta	T11	NR	E	Y	N	E
Dagenais et al. 2006	36	F	IT	Thoracic aorta	T9	NR	A (T9)	EDV	Y	A (T9)
Chock et al. 2014	25	M	P	Thoracic aorta	T10–T11	C	A (T10)	EDV	Y	A (T10)
Cultrera et al. 2017	41	M	O/N BF	Thoracic aorta	T6–T7	C	E	EDV	Y	E
Kovari et al. 2017	31	F	P	Thoracic aorta	T8–T9	B	E	EDV	Y	E
Santoro et al. 2018	46	M	IT	Thoracic and abdominal aorta	T11–T12	C	A (T11)	N	Y	A (T11)
Erdogan et al. 2021	49	M	BF	Thoracic aorta	T11	NR	NR	EDV	Y	NR
Mohammed et al. 2021	35	M	IT	Thoracic aorta	T11	NR	A (T11)	Y	Y	A (T11)
Present case	45	M	O/N BF	Thoracic aorta	T10	B	E	EDV	Y	E

IT, intimal tear; P, pseudoaneurysm; O/N, obstruction/narrowing; BF, direct lesion by bone fragment; EDV, endovascular; NR, not reported; Y, yes; N, no.

## Case report

**Figure 1:**
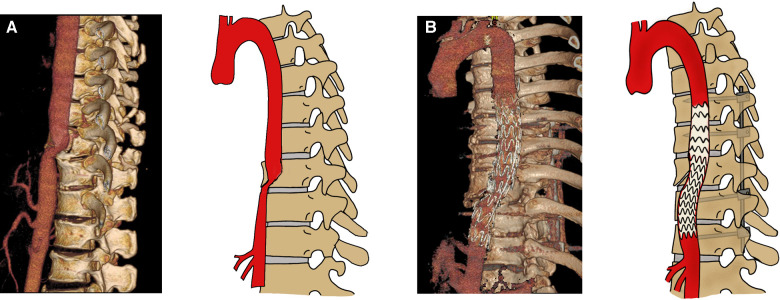
Computed tomography angiograms obtained before (**A**) and after (**B**) endovascular repair of the thoracic aortic injury and fixation of the spinal fractures.

**Figure 2:**
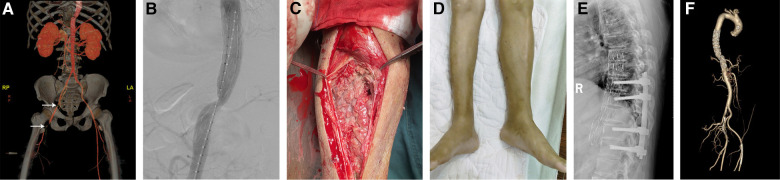
Filling defect at the right external iliac artery and femoral artery bifurcation with severe luminal stenosis (white arrows) (**A**). Angiogram obtained on completion of the procedure showing adequate placement of the stent graft with no endoleak or kink (**B**). Thirteen days after the right lower leg fasciotomy, there was still extensive necrosis in the posterior leg muscles (**C**). The circumference of the right calf was smaller than that of the left calf at 7 months after flap reconstruction surgery (**D**). A lateral radiograph obtained after 1 year of follow-up shows the aortic stent graft *in situ* and stabilization of the vertebral fracture at T10 (**E**). A repeated CTA performed 1 year of follow-up showed the stent without endoleak (**F**).

The case was a 45-year-old man who was taken to a local community hospital on January 6, 2021 after falling backwards from a height of nearly 6 meters and landing on his back. On arrival, he had severe pain in the back, chest, and abdomen with numbness in the lower limbs. The patient’s past medical history included hypertension, gout, and moderate obesity. Computed tomography (CT) scans showed an AO type B3 injury at T10 and a right-sided pneumothorax. Bilateral hemopneumothorax was drained at that time. One day later, he was transferred to our hospital. Physical examination revealed tenderness at T9–T11, typical limitation of activity in the right lower limb, no pulsation in the right dorsalis pedis artery, and increased pressure in the right lower limb. There were no pathological signs. CT of chest showed multiple rib fractures on both sides, a right hemopneumothorax, and left pleural effusion. Contrast-enhanced CT scans of the abdomen and pelvis showed lacerations to the liver and spleen and that the T10 vertebra was adjacent to the thoracic aorta. B-scan ultrasonography indicated embolism of the right femoral artery. CTA of the thoracic aorta also documented the vertebral fracture at T9–11, which was classified as Magerl type B3; Figure 1A), luminal stenosis in the thoracic aorta and tortuosity at the level of T10, and a filling defect at the junction of the external iliac and common femoral arteries with severe luminal stenosis (Figure 2A). The serum alanine aminotransferase (ALT) level was elevated at 3028 U/l, the serum creatinine level was 308 µmol/l, and the serum D-dimer level was >20 mg/l.

**Figure 3:**
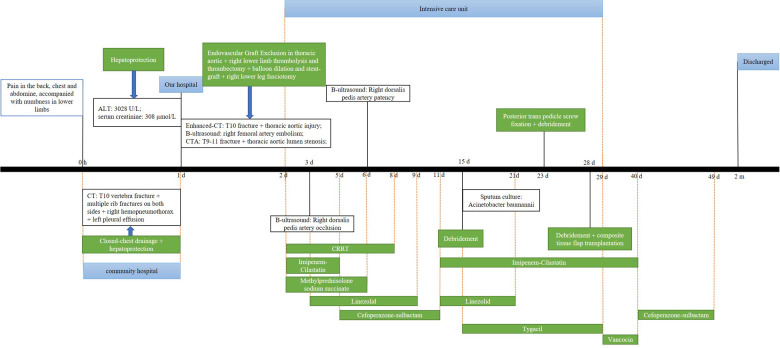
Timeline showing the diagnosis, treatment, and outcome in our patient. CT, computed tomography; CRRT, continuous renal replacement therapy; ALT, alanine aminotransferase; CTA, computed tomography angiography.

Based on the clinical and physical findings, the final diagnoses were vertebral fracture combined with thoracic aortic injury, deep venous thrombosis in the right lower limb, periosteal compartment syndrome in the right lower leg, multiple rib fractures on both sides, right hemopneumothorax, left pleural effusion, hepatic and renal damage, and hypertension. For the patient in a critical condition, an emergency procedure consisting of endovascular graft exclusion in the thoracic aorta + right lower limb thrombolysis and thrombectomy + balloon dilation and stent grafting was performed. Severe luminal stenosis was seen in the aorta at the level of T10. An Ankura endovascular stent (XJZDZ30-26-160; Lifetech Scientific, Shenzhen, China) was introduced, with the distal end positioned above the celiac trunk and the proximal end positioned distal to the left subclavian artery (vertebral levels at T6-T12). Although the stent was successfully delivered, there was still relatively heavy stenosis locally. A balloon (12 mm × 80 mm; BSX, USA) was expanded at the site of the stenosis and the stent-graft placement was considered to be adequate with no endoleak or kink (Figure 2B). Angiography showed filling of the defect well beyond the bifurcation of the right femoral artery. Therefore, the rigid guide wire was exchanged for an AngioJet thrombectomy catheter, which was introduced into the popliteal artery segment and advanced into the femoral artery for thrombectomy. The total thrombectomy time was 210 s. The proximal part of the bifurcation was still not visualized on repeat angiography. Although the segment between the superficial distal end of the femoral artery and the popliteal artery was well visualized, the area below the knee was not. The locally severe stenosis extended to the distal end of the posterior tibial artery; therefore, a balloon catheter was introduced to the distal end of this vessel. The posterior tibial artery was expanded from the distal end to the proximal end, with expansion continued to the distal end of the popliteal artery. Repeat imaging of the posterior tibial artery showed that the anterior tibial and peroneal arteries were both occluded. An 8 mm × 80 mm Ankura, endovascular stent was introduced and successfully deployed in the poorly visualized portion of the femoral artery, and blood flow in the stent was confirmed to be adequate on repeat imaging. Angiography at the left puncture site showed luminal stenosis in the left external iliac artery segment. Considering the possibility of intimal injury, another endovascular stent (8 mm × 100 mm, W.L. Gore / Associates, Flagstaff, AZ, USA) was implanted and deployed in the left external iliac artery segment. Angiography confirmed adequate blood flow through the stent. Physical examination showed obvious ecchymosis, swelling, and high pressure in the patient’s right calf. The musculoskeletal physician considered that incision was necessary for decompression, and a right lower leg fasciotomy was performed. The tibialis anterior, gastrocnemius, and soleus muscles were ischemic and dark purple in color. Continuous renal replacement therapy was administered to reduce the amount of toxins released by the necrotic muscle tissue in the right lower limb. Supportive treatment, including hepatoprotection, anticoagulation, and antibiotic therapy, was also started. The patient subsequently underwent two debridement procedures, flap reconstruction surgery on his right lower leg, and posterior transpedicular screw fixation at T9–T11 for his vertebral fracture at T10 (Figure 1B). The posterior leg muscles were extensively necrotic at the time of the first surgical debridement (Figure 2C). The timeline of treatment is shown in Figure 3.

**Figure 4:**
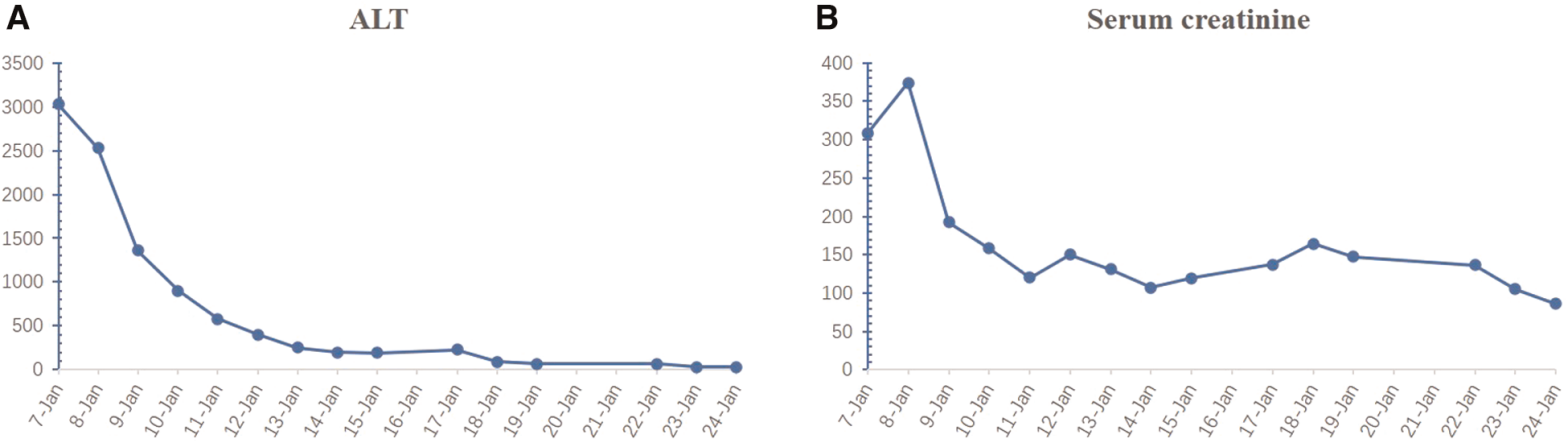
Changes in the ALT (**A**) and serum creatinine (**B**) levels over time. ALT, alanine aminotransferase.

The ALT and serum creatinine levels decreased with supportive treatment (Figure 4). Muscle power was grade 0/5 below the right knee and grade 5/5 in the other limbs at the time of discharge from hospital. We asked the patient to review the arterial CTA and x-ray during the follow-up to find out whether the patient’s stent had endoleak or collapsed. Seven months later, follow-up clinical and radiological examinations confirmed that there was no further damage to the right lower limb (Figure 2D). Repeat x-ray and CTA performed 1 year after the patient was discharged showed the stent was stable and without endoleak (Figures 2E,F). The patient is currently able to walk and perform physical work normally without pain in the back and lower extremities.

## Discussion

Traumatic aortic injury occurs in response to high-energy forces and is likely to be fatal if untreated (12). The aortic isthmus is the area most commonly affected because its position is relatively fixed in relation to the aortic arch and descending aorta (13). When high-energy force is applied, inertial swing occurs between the aortic arch and descending aorta, and the resulting shear force causes damage to the aortic isthmus. This mechanism of injury is most common in motor vehicle accidents followed by falls from height (14, 15). Damage to the thoracic or abdominal aorta is rare (16). The incidence of aortic injury in patients with lumbar burst fracture is 11.3%, and the probability of a missed diagnosis in these patients is reportedly as high as 78% (17). CTA has been considered the gold standard for evaluation of patients suspected to have aortic injury (18) and allows adequate investigation of the extent of vascular injury (11). In our case, the attending clinician at the referring hospital could not detect the damage to the aorta on CT, leading to delayed diagnosis and treatment. When the patient was transferred to our hospital, contrast-enhanced CT showed that the thoracic aorta was adjacent to the thoracic vertebral fracture, and CTA was performed to evaluate the status of the aorta. Fractures of the spine accompany a large proportion of thoracic aorta injuries, which may manifest as aortic rupture with a pre-hospital mortality rate of up to 85% (7), pseudoaneurysm formation, or delayed bleeding, which is potentially life-threatening. Therefore, CTA should be performed as early as possible in patients suspected to have an aortic injury. If an aortic lesion is confirmed and the patient’s condition permits, the treatment plan should be determined as rapidly as possible (7, 19). In this situation, spinal surgery could be hazardous if performed before the vascular repair. Santoro et al. reported that 60% of patients who were treated in this order did not survive (2). Open surgical repair *via* thoracotomy has been the gold standard treatment for aortic injury combined with spinal fracture but has many potential complications, including renal failure, respiratory dysfunction, spinal cord ischemic injury, and pneumonia (8). Moreover, these patients usually present with multiple trauma and are seriously unwell. Open surgical repair may not be the best treatment for these patients in view of the lengthy operation time, high surgical risk, and surgical invasiveness. Many patients have difficulty tolerating this type of surgical procedure, and thoracotomy requires placement of the patient in the lateral decubitus position, which may destabilize the spine further, thereby worsening a severe aortic injury and spinal cord damage (20, 21). The advent of TEVAR means that it is now possible to treat patients with aortic injury combined with spinal fractures. The advantages of TEVAR are its minimal invasiveness, the ability to treat the patient in the supine decubitus position, a shorter operation time, a shorter postoperative hospital stay with reduced costs, a lower rate of complications (renal failure, spinal cord injury, pneumonia, respiratory failure) that result in a prolonged stay in the intensive care unit, and less need for red blood cell transfusion (22, 23). Furthermore, TEVAR may be associated with less intraoperative and in-hospital mortality and a lower risk of paraplegia (24). However, there are some challenges when TEVAR is used to treat blunt thoracic aortic injury. First, the reduced diameter of the aorta near the injury results in the stent potentially being too large, which may lead to serious complications, such as stent infolding or malapposition and possibly stent displacement or collapse (25). Second, there are risks of stent fracture, material fatigue, and endoleak in younger patients who undergo TEVAR. The incidence of endoleak is up to 14.4% after placement of an aortic stent (26). Although there has been a decrease in the incidence of endoleak with technological advances, the risk of endoleak is lifelong in patients who undergone TEVAR and ongoing follow-up is required (26). Therefore, open repair may have medium-term to long-term advantages in younger low-risk patients. Once the aortic surgery is completed, the spinal fractures can be repaired in the prone decubitus position.

In our case, contrast-enhanced CT suggested injury to the thoracic aorta. Considering that the upper and lower vascular walls were uninterrupted with no extravasation of contrast agent and the patient’s vital signs were stable, we did not perform emergency surgery. However, when the blood supply to the patient’s right lower limb deteriorated, B-scan ultrasonography indicated arterial embolism, and CTA indicated thoracic aortic injury, we operated immediately. The patient underwent TEVAR as a minimally invasive treatment to ensure the patency of blood vessels, reduce further damage to the body, and allow internal fixation of the thoracic vertebral fracture as a second stage. Prolonged compression of thoracic aortic result in liver and kidney damage, andthese function improved gradually on other adjuvant therapy. Unfortunately, the patient developed extensive muscle necrosis in the right lower extremity as a result of the prolonged ischemia.

Including our case, a total of 10 cases could be reviewed. The mean age was 36 years (range, 21–49). Four cases had neurological impairment (grade A, American Spinal Injury Association Impairment Scale), five had normal neurological status, and neurological status was not reported for one case. The vascular damage was described as follows: intimal tear (*n* = 4, 40%), pseudoaneurysm (*n* = 3, 30%), obstruction or narrowing (*n* = 2, 20%), and a lesion caused directly by a bone fragment (*n* = 1, 10%). The vascular injury was repaired surgically in 9 cases (90%) and before spinal surgery in all cases. Open surgical repair was performed in 3 cases, TEVAR in 6 cases, and conservatively in 1 case. Hemodynamic stability was strictly maintained during thoracic spine surgery in the patient with aortic injury who was treated conservatively. Neurological outcomes were reported for 9 patients, and were unaffected in all cases. Review of the results in these cases indicates that it is safe and reliable to repair the vascular injury first and then proceed to surgical management of vertebral fractures when the patient’s hemodynamic status has stabilized. The decision regarding whether to perform open surgery or TEVAR for the aortic injury should be based on surgical risk, age, and the severity of injury.

This case has several limitations. First, although the patient presented with multiple trauma, thoracic CT performed at the referring hospital did not detect the aortic injury. Prolonged compression of the thoracic aorta led to local dissection, deep venous thrombosis in the right lower limb, and right lower leg periosteal compartment syndrome in the right lower leg. The ensuing impairment of liver function and extensive necrosis of muscles and nerves below the right knee joint resulted in physical disability with cost implications for the patient. Second, delayed diagnosis and treatment led to a prolonged stay in the intensive care unit with onset of bacteremia. Third, generation stent grafting is possible when the thoracic aorta is compressed by a thoracic vertebral fracture. The main limitation of our literature review is that it is based on only 9 reported cases in addition to our present case. Therefore, more cases need to be accumulated in the future.

## Conclusion

Delayed diagnosis of aortic injury can lead to serious complications. Prolonged compression can result in liver and kidney damage, venous thrombosis in the lower extremities, and ischemic necrosis in muscle tissue. While the long-term outcomes of TEVAR are unknown in this patient population, TEVAR has certainly improved the outcome sin these patient as compared to open surgery at least as far as survival is considered. The long-term possible sequalae of stenting can be dealt in a more stable patient.

## References

[1] Inaba K Kirkpatrick AW Finkelstein J Murphy J Brenneman FD Boulanger BR Blunt abdominal aortic trauma in association with thoracolumbar spine fractures. Injury. (2001) 32(3):201–7. 10.1016/S0020-1383(00)00203-5 11240295

[2] Santoro G Ramieri A Chiarella V Vigliotta M Domenicucci M. Thoraco-lumbar fractures with blunt traumatic aortic injury in adult patients: correlations and management. Eur Spine J. (2018) 27(Suppl 2):248–57. 10.1007/s00586-018-5601-5 29663146

[3] Vargo PR Maigrot JL Roselli EE. Chronic thoracoabdominal aortic dissection: endovascular options to obliterate the false lumen. Ann Cardiothorac Surg. (2021) 10(6):778–83. 10.21037/acs-2021-taes-23 34926180 PMC8640879

[4] Poelaert J Defreyne L Vermassen F Colardyn F. Post-traumatic transverse dissection of the caudal thoracic aorta diagnosed by transoesophageal echocardiography. Eur J Emerg Med. (1995) 2(2):105–7. 10.1097/00063110-199506000-00011 9422193

[5] Murakami R Tajima H Ichikawa K Kobayashi Y Sugizaki K Yamamoto K Acute traumatic injury of the distal descending aorta associated with thoracic spine injury. Eur Radiol. (1998) 8(1):60–2. 10.1007/s003300050339 9442131

[6] Dagenais F Nadeau G Normand JP Turcotte R Mathieu P Voisine P. Endovascular treatment of a blunt thoracic aortic laceration due to thoracolumbar spine fracture. Innovations. (2006) 1(3):123–5. 10.1097/01243895-200600130-00006 22436647

[7] Chock MM Aho J Naik N Clarke M Heller S Oderich GS. Endovascular treatment of distal thoracic aortic transection associated with severe thoracolumbar spinal fracture. Vascular. (2015) 23(5):550–2. 10.1177/1708538114560458 25406266

[8] Cultrera F Gamberini E Iacono G Turicchia GU Agnoletti V Tosatto L. Unstable thoracic spine fracture with aortic encroachment: a potentially fatal association and a suggested treatment. Int J Surg Case Rep. (2017) 39:181–4. 10.1016/j.ijscr.2017.08.015 28846951 PMC5573841

[9] Kovari VZ Pecsi F Cs Janvari M Veres R. Initial experience with the treatment of concomitant aortic pseudoaneurysm and thoracolumbar spinal fracture: case report. Trauma Case Rep. (2017) 12:48–53. 10.1016/j.tcr.2017.10.019 29644285 PMC5887094

[10] Erdogan KE Besler MS Canyigit M Hidiroglu M. Endovascular repair of a distal thoracic aortic transection in association with traumatic burst fracture. Indian J Thorac Cardiovasc Surg. (2021) 37(5):554–7. 10.1007/s12055-021-01138-9 34511763 PMC8387510

[11] Mohammed AA Shulaiba FR Alhety MHI Al Saadi HSAH El Yafawi B. Aortic impingement in displaced traumatic spine fracture with complete spinal cord transection: a case report. Dubai Medical Journal. (2021) 4(4):358–64. 10.1159/000520129

[12] Meyer CC Engelbrecht A. Traumatic aortic dissection presenting with respiratory arrest. Afr J Emerg Med. (2015) 5(1):e5–7. 10.1016/j.afjem.2014.10.007

[13] McNamee CJ Knight JL. Blunt traumatic avulsion of an intercostal artery: an unusual case of thoracic aortic injury. Can J Surg. (1992) 35(6):658–60. 1458395

[14] Demetriades D Velmahos GC Scalea TM Jurkovich GJ Karmy-Jones R Teixeira PG Operative repair or endovascular stent graft in blunt traumatic thoracic aortic injuries: results of an American association for the surgery of trauma multicenter study. J Trauma. (2008) 64(3):561–70; discussion 570–561. 10.1097/TA.0b013e3181641bb3 18332794

[15] Reisman JD Morgan AS. Analysis of 46 intra-abdominal aortic injuries from blunt trauma: case reports and literature review. J Trauma. (1990) 30(10):1294–7. 10.1097/00005373-199010000-00017 2213938

[16] Fabian TC Richardson JD Croce MA Smith JS Jr. Rodman G Jr. Kearney PA Prospective study of blunt aortic injury: multicenter trial of the American association for the surgery of trauma. J Trauma. (1997) 42(3):374–80; discussion 380–373. 10.1097/00005373-199703000-00003 9095103

[17] Deng H Tang T-X Tang L-S Chen D Luo J-L Dong L-M Thoracic spine fractures with blunt aortic injury: incidence, risk factors, and characteristics. J Clin Med. (2021) 10(22):5220. 10.3390/jcm10225220 34830504 PMC8623488

[18] White CS Mirvis SE. Pictorial review: imaging of traumatic aortic injury. Clin Radiol. (1995) 50(5):281–7. 10.1016/S0009-9260(05)83417-8 7743715

[19] Domenicucci M Ramieri A Landi A Melone AG Raco A Ruggiero M Blunt abdominal aortic disruption (BAAD) in shear fracture of the adult thoraco-lumbar spine: case report and literature review. Eur Spine J. (2011) 20 (Suppl 2):S314–319. 10.1007/s00586-011-1732-7 21380748 PMC3111528

[20] Decker S Omar M Krettek C Müller CW. Elective thoracotomy for pedicle screw removal to prevent severe aortic bleeding. World J Clin Cases. (2014) 2(4):100–3. 10.12998/wjcc.v2.i4.100 24749121 PMC3985037

[21] Watanabe K Yamazaki A Hirano T Izumi T Sano A Morita O Descending aortic injury by a thoracic pedicle screw during posterior reconstructive surgery: a case report. Spine. (2010) 35(20):E1064–1068. 10.1097/BRS.0b013e3181ed29c1 20802385

[22] Tang GL Tehrani HY Usman A Katariya K Otero C Perez E Reduced mortality, paraplegia, and stroke with stent graft repair of blunt aortic transections: a modern meta-analysis. J Vasc Surg. (2008) 47(3):671–5. 10.1016/j.jvs.2007.08.031 17980541

[23] Brand S Breitenbach I Bolzen P Petri M Krettek C Teebken O. Open repair versus thoracic endovascular aortic repair in multiple-injured patients: observations from a level-1 trauma center. Arch Trauma Res. (2015) 4(4):e27183–e27183. 10.5812/atr.27183 26848470 PMC4733514

[24] Riesenman PJ Farber MA Rich PB Sheridan BC Mendes RR Marston WA Outcomes of surgical and endovascular treatment of acute traumatic thoracic aortic injury. J Vasc Surg. (2007) 46(5):934–40. 10.1016/j.jvs.2007.07.029 17980280

[25] Agostinelli A Carino D Borrello B Marcato C Volpi A Gherli T Blunt traumatic injury to the thoracic aorta treated with thoracic endovascular aortic repair: a single-centre 20-year experience. Interact Cardiovasc Thorac Surg. (2019) 28(1):17–22. 10.1093/icvts/ivy211 30007311

[26] Brenner M Teeter W Hadud M Hoehn M O’Connor J Stein D Long-term outcomes of thoracic endovascular aortic repair: a single institution's 11-year experience. J Trauma Acute Care Surg. (2017) 82(4):687–93. 10.1097/TA.0000000000001365 28129260

